# Exploring the ageing methylome in the model insect, *Nasonia vitripennis*

**DOI:** 10.1186/s12864-024-10211-7

**Published:** 2024-03-22

**Authors:** K. Brink, C. L. Thomas, A. Jones, T. W. Chan, E. B. Mallon

**Affiliations:** 1 Department of Genetics and Genome Biology, University of Leicester, University Road, Leicester, UK; 2 Institute for Evolution and Biodiversity, University of Muenster, Huefferstrabe, Muenster, Germany; 3 School of Life Sciences, Gibbet Hill Campus, The University of Warwick, Coventry, UK

**Keywords:** Epigenetics, BS-seq, Hymenoptera, Epigenetic clock, Entropy

## Abstract

**Background:**

The ageing process is a multifaceted phenomenon marked by the gradual deterioration of cellular and organismal functions, accompanied by an elevated susceptibility to diseases. The intricate interplay between genetic and environmental factors complicates research, particularly in complex mammalian models. In this context, simple invertebrate organisms have been pivotal, but the current models lack detectable DNA methylation limiting the exploration of this critical epigenetic ageing mechanism.

This study introduces Nasonia vitripennis, the jewel wasp, as an innovative invertebrate model for investigating the epigenetics of ageing. Leveraging its advantages as a model organism and possessing a functional DNA methylation system, Nasonia emerges as a valuable addition to ageing research.

**Results:**

Whole-genome bisulfite sequencing unveiled dynamic alterations in DNA methylation, with differentially methylated CpGs between distinct time points in both male and female wasps. These changes were associated with numerous genes, enriching for functions related to telomere maintenance, histone methylation, and mRNA catabolic processes. Additionally, other CpGs were found to be variably methylated at each timepoint. Sex-specific effects on epigenetic entropy were observed, indicating differential patterns in the loss of epigenetic stability over time. Constructing an epigenetic clock containing 19 CpGs revealed a robust correlation between epigenetic age and chronological age.

**Conclusions:**

*Nasonia vitripennis* emerges as a promising model for investigating the epigenetics of ageing, shedding light on the intricate dynamics of DNA methylation and their implications for age-related processes. This research not only expands the repertoire of ageing models but also opens avenues for deeper exploration of epigenetic mechanisms in the context of ageing.

**Supplementary Information:**

The online version contains supplementary material available at 10.1186/s12864-024-10211-7.

## Introduction

Ageing is a complex biological process characterized by a progressive decline in cellular and organismal function, accompanied by an increased susceptibility to diseases. Ageing is influenced by many environmental and genetic components. The effects of these components influence each other making them difficult to investigate, especially in complex mammalian models. Therefore, a large body of ageing research is based on simple invertebrate model organisms [1, 2]. Advantages include easy and cheap to keep in a laboratory, short life span, genetic and molecular tools available and a sequenced genome. However, the current invertebrate models of ageing (Drosophila [3] and *C. elegans* [4]) do not possess detectable DNA methylation.

DNA methylation involves the addition of a methyl group to cytosine residues, predominantly occurring at cytosine-phosphate-guanine (CpG) dinucleotides. Studies have consistently demonstrated dynamic changes in DNA methylation patterns during ageing, influencing gene expression, cellular function, and ultimately contributing to the ageing phenotype [5]. Despite not being found in our current two invertebrate models of ageing, this is not true universally in invertebrates and changes in DNA methylation have been associated with ageing in ants [6], bumblebees [7] honeybees [8] and the crustacean *Daphnia magna* [9]. Although social insects present an almost unique opportunity for an invertebrate model of the effects of social environment on ageing [10], their colony structure prevent them from being easily kept general models of ageing. Although *Daphnia magna* is relatively easy to keep, as a crustacean we can not take advantage of the large amount of research on hymenopteran DNA methylation [11].

We propose the jewel wasp, *Nasonia vitripennis* as a general model for epigenetic ageing as it possesses both the advantages of a model systems mentioned above [12] and has a functional methylation system [13, 14]. DNA methylation has been shown to be vital for *Nasonia* development [15]. It has also been shown to be invovled in a number of *Nasonia* phenotypes including diapause [16] and sex allocation [17]. Ageing in *Nasonia* has been studied with reference to lipogenesis [18], diet [19], ploidy [20], diapause [21] and host species [22]. To our knowledge, the changes in DNA methylation as *Nasonia* ages have never been measured. Here, we establish *Nasonia* as a model to investigate the epigenetics of ageing by first measuring its methylome as it ages.

The omics era has seen a rapid growth in studies measuring these dynamic changes in DNA methylation during ageing. They are usually based on whole genome bisulphite sequencing (WGBS) [23]. Perhaps the most obvious way to search for patterns in this WGBS data are differentially methylated positions (DMPs). These are characterised by changes in average DNA methylation levels across individuals or tissues at single base positions (CpGs). These CpGS either become more methylated as an organism ages (hypermethylation) or less methylated (hypomethylation). These DMPs represent single base pair changes in DNA methylation as the organism ages.

Epigenetic drift is the increased variability in the epigenome found through the course of an individual’s life caused by the accumulation of mistakes in preserving epigenetic patterns. Epigenetic drift leads to a decrease in the body’s ability to maintain homeostasis [24]. This can be measured in at least two ways. Variably methylated positions (VMPs) rather than comparing the average methylation at a CpG over time, measures how variable the methylation at a site is as organisms age. An alternative measure of epigenetic drift is Shannon’s entropy, i.e. the loss of information in the whole epigenome over time [25]. An increase in entropy means the epigenome is becoming less predictable, that is more variable over time.

An epigenetic clock is an emergent property of the DNA methylation status of a large number of genes, calculated using supervised machine learning methods [25–27]. There is evidence epigenetic age mirrors true biological age and its associated morbidity and mortality better than chronological age [5]. However, their utility as measures of changes in biological age for clinical interventions is limited as their mechanistic basis is not understood [28].

**Fig. 1:**
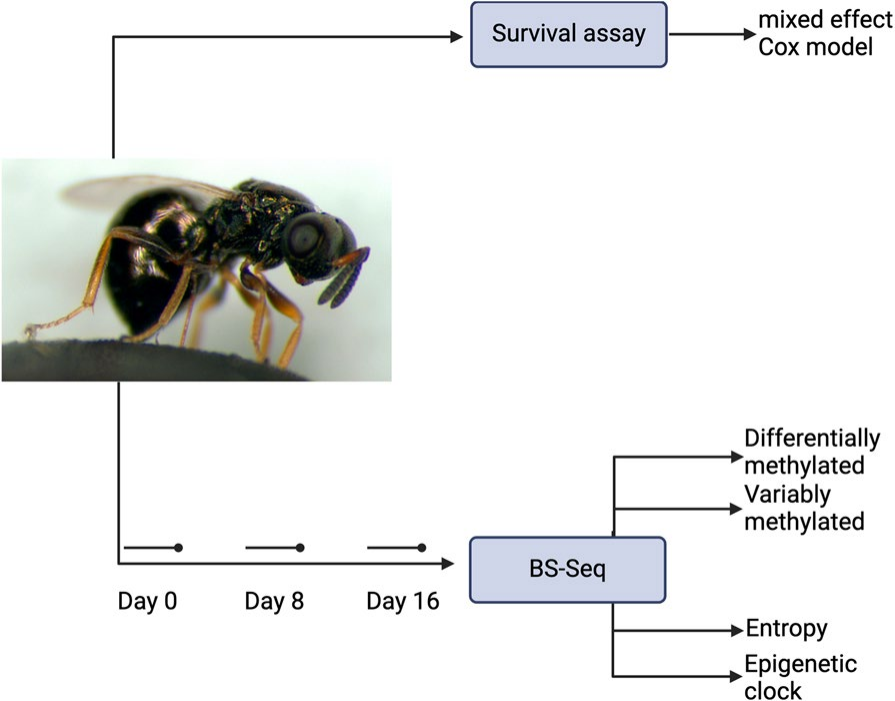
Schematic overview of experimental design with study organism, *Nasonia vitripennis*. Image credit: M.E. Clark public domain

In this paper, we measure chronological ageing and changes in the methylome using whole genome bisulfite sequencing (WGBS) (Fig. 1) in order to discover if *Nasonia vitripennis*, unlike the other two invertebrate models of ageing possesses an ageing methylome.

## Methods

### Life span

*Nasonia* were of the *Nasonia vitripennis* species from the Leicester strain which has been kept for over eight years and originated from the AsymC strain. Wild-type wasps were maintained at 25^∘^C, 40% humidity in a 12-h dark/light cycle. Adults, within 24 hours of eclosion, were placed in tubes of ten single-sex individuals. They were fed 20% sucrose *ad libitum*, refreshed daily. These were checked every day for survival. 70 females and 67 males were used. A mixed effect Cox model treating tube as a random effect was implemented using the survival package (v.3.4) [29] and coxme package (v.2.2) [30] in R 4.2.2 [31].

### DNA extraction

Wasps were collected within twenty four hours of eclosion. Some females may have been mated as they were allowed to mix with males for upto the first fifteen hours after eclosion. Wasps were then collected under light CO_2_ anesthesia and placed into single-sex vials containing ten individuals. They were then provided with filter paper soaked in 20% sucrose which was changed daily. Day zero wasps were collected at the end of the first twenty four hours after eclosion then samples on the eighth and sixteenth day. Thirty wasps of each sex were used for each time-point. Ten wasps from the same sex were pooled for each replicate, creating three replicates for each sex at each time point. Therefore we had three replicates for males and three replicates for females at each of day zero, eight and sixteen after eclosion. Wasps were immediately frozen in liquid nitrogen and stored at minus 80^∘^C freezer for sequencing. DNA was extracted using Qiagen’s DNAeasy Blood and Tissue kit. DNA quality was assessed by NanoDrop 2000 spectrophotometer (Thermo Scientific), 1% agarose gel and Qubit (dsDNA BR Assay, ThermoFisher).

### Whole genome bisulfite sequencing

WGBS sequencing was carried out by BGI Tech Solution Co., Ltd.(Hong Kong). A 1% unmethylated lambda spike was included in each sample in order to assess bisulfite conversion rates. For WGBS samples, library quality was checked with FastQC (v.0.11.5; [32]). One day eight male library appeared to be mislabelled by the sequencing company. This was not included in further analysis to prevent any uncertainty. Therefore for the BS-seq analysis we had three replicates for males and three replicates for females at each of day zero and sixteen after eclosion but three replicates for males and two replicates for females at day eight. Paired-end reads were aligned to the *Nasonia vitripennis* reference genome (Nvit_PSR1.1, Refseq accession no. GCA_009193385.1, [33]) using the Bowtie 2 aligner (v.2.2.9; [34]) within the Bismark software (v.0.18.1; [35]) under standard parameters. Samples sequenced across multiple files were merged using samtools (v.1.9; [36]). Files were deduplicated using Bismark, and methylation counts were extracted in different contexts using the bismark_methylation_extractor command (v.0.18.1; [35]). Destranding was carried out using the coverage2cytosine script from Bismark using the merge_CpG command to increase coverage by pooling the top and bottom strand into a single CpG [35]. Reads were also aligned to the unmethylated lambda reference genome to calculate the error rate of the C-T conversion (Refseq accession no. GCF 000840245.1).

### Differential methylation analysis

Output from the coverage2cytosine script was then inputted into the R package methylKit (v.3.14; [37]) where files were filtered and normalised based on coverage, removing sites with abnormally high coverage (greater than 99% percentile) or with a coverage less than ten in each sample.

A binomial test was then applied to the filtered CpG sites where the lambda conversion rate was used as the probability of successes and a false discovery rate (FDR) of *p* < 0.05 [38]. As the majority of sites in the *Nasonia* genome show zero methylation, only CpGs which were methylated in at least one sample were retained. On these methylated CpGs, differential methylation analysis was performed using the calculateDiffMeth command in methylKit, which implements a logistic regression model. Differentially methylated CpG sites were classed as having a minimum difference of > 15% methylation and a *q*-value < 0.05. Differential methylation analyses were performed across age in each sex.

Genes were classed as differentially methylated if they contained at least two differentially methylated CpG and a minimum weighted methylation difference of 15% across the entire feature [39]. Weighted methylation level is classed as the total number of methylated cytosines (C) within a region (i), divided by the total coverage of that region [39]. A mixed effect model examined the effect of sex and age on this weighted methylation with CpG as a random effect.

### Variable methylation analysis

Variable methylation analysis was carried out on the methylated cytosines. A beta regression model was applied, with methylation proportion as the response variable, and chronological age and sex as predictor variables for each cytosine. The beta regression was implemented using the betareg function from the R package betareg (version 3.1.4) [40].

To identify Variable Methylated Positions (VMPs), the Breusch-Pagan test for Heteroscedasticity was performed using the bptest function in the R package lmtest (version 0.9.40) [41]. Multiple testing corrections were applied using the Holm-Bonferroni method. CpG sites demonstrating significant heteroscedasticity were classified as age-related VMPs.

### Epigenetic drift

Entropy is calculated as;

1 $$\begin{aligned} Entropy = \frac{1}{N*\log \frac{1}{2}}\sum \limits _i[{MF_i * \log {MF_i} + (1-MF_i) * (1- \log {MF_i})}] \end{aligned}$$

with $MF_i$ the fraction of methylation on a given CpG and N the total number of CpGs measured (5290 significantly differentially methylated CpGs).

The effects of chronological age and sex on entropy were analysed using a beta regression using the betareg package in R [42]. Post-hoc tests were carried out using the emmeans package in R [43].

### Elastic net regression

Chronological age was regressed against the 5290 age significant CpGs’ beta values using an elastic net regression with a 3-fold cross validation implemented in the glmnet R package [44]. The reported accuracy of the epigenetic clock is expected to be overly optimistic since the regression model used cytosines that relate to age in the entire data set. The elastic net regression identified 19 CpGs that predict age. The epigenetic age of each replicate is predicted based on these CpGs methylation state. This epigenetic age was correlated with chronological age using a spearman’s rank correlation. Age acceleration was calculated as the residual from regressing chronological age against epigenetic age [27].

### Gene ontology enrichment

Gene ontology (GO) terms for *N. vitripennis* were taken from the ensembl metazoa database [45]. GO enrichment analysis was carried out using the hypergeometric test with Benjamini-Hochberg [46] multiple-testing correction, q <0.05. GO terms from variably methylated genes and epigenetic clock genes were tested against a GO term database made from the GO terms associated with all methylated genes. Genes were determined as methylated if they had a mean weighted methylation level taken across all replicates greater than the lambda spike weighted methylation level of 0.05 in any of the samples within each comparison. Genes which were consistently hyper or hypo methylated in males or females were tested against all differentially methylated genes in any comparison. REVIGO [47] was used to generate treemaps.

## Results

### Survivourship analysis

**Fig. 2:**
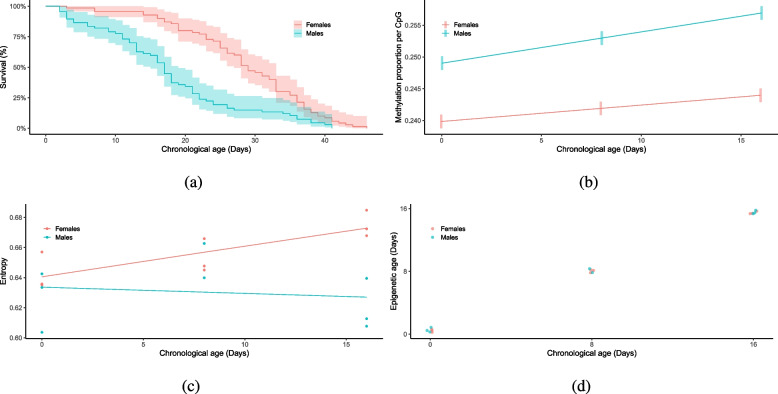
**a** Kaplan-Meier survival curves for female (n = 70) and male (n = 67) *Nasonia vitripennis* adults. Shaded areas represent the 95% confidence intervals. **b** Estimated marginal means of methylation proportion per methylated CpG against chronological age. Shaded bars are 95% confidence intervals **c** Scatterplots of epigenetic entropy based on the 5290 age-related differentially methylated CpGs over time for male and female *Nasonia*. Each point is a single WGBS library made up of ten individuals. Lines represent the beta regression predictions **d** Scatterplot of epigenetic age versus chronological age of male (*n*=8) and female (*n* = 9) *Nasonia* samples. Each sample is made up of the whole bodies of ten individuals

Males and females showed different patterns of life expectancy (Cox mixed-effects model: Hazard ratio for females = 2.48 (standard error of coefficient = 0.233), z = 3.89, *p* = $9.8\times 10^{-5}$), with females’ mean life expectancy being 29 days and males’ being 17 days, see Fig. 2a. This was greater than the 16.6 days for females and 10.7 days for males previously found for sucrose-fed individuals [48].

### Differential methylation analysis

Five thousand two hundred ninety CpGs were found to be significantly differentially methylated between at least two time points in males or females. Of these 48% were hypomethylated and 52% hypermethylated. Overall, DNA methylation increases in Nasonia as it ages with males having higher levels (mixed effect model: $\chi ^2$ = 933.64, d.f. =1, *p* < $2.2\times 10^{-16}$) (Fig. 2b). These CpGs were found in 2295 annotated genes (Supplemental Table S1). As males aged, 280 CpGs were hypermethylated consistently, corresponding to 114 annotated genes (Supplemental Table S2). 218 CpGs were consistently hypomethylated as males age (83 genes - Supplemental Table S3). For females, 58 CpGs (26 genes - Supplemental Table S4) were consistently hypermethylated and 34 CpGs (14 genes - Supplemental Table S5) hypomethylated. There was no overlap between these groups of CpGs.

In order to explore the function of these genes, we carried out a GO enrichment analysis of the four groups of consistently methylated genes compared to all significantly differentially methylated genes. Genes which are consistently hypermethylated as males age are enriched for telomere function including “*regulation of telomere maintenance*” (GO:0032204), “*telomere organization*” (GO:0032200) and “*meiotic attachment of telomere to nuclear envelope*” (GO:0070197) (Supplemental Fig. S1). Genes which are consistently hypomethylated as males age are enriched for GO terms associated with histone methylation including “*regulation of histone methylation*” (GO:0031060) and “*histone H3-K4 trimethylation*” (GO:0080182). “*response to ecdysone*” (GO:0035075) was also enrich in hypomethylated genes in ageing males (Supplemental Fig. S2). Genes which are consistently hypermethylated as females age are enriched for “*nuclear-transcribed mRNA catabolic process*” (GO:0000956), see Supplemental Fig. S3. Genes which are consistently hypomethylated as females age are enriched for “*negative regulation of JUN kinase activity*” (GO:0043508) and “*protein localization to kinetochore*” (GO:0034501), see Supplemental Fig. S4.

### Variably methylated positions

Twenty-nine thousand twelve age-related VMPs were found amongst the 375,403 methylated CpGs. The identified VMPs were located at 2,451 different genes (Supplemental Table S6). Supplemental Fig. S5 shows a treemap of GO terms associated with these genes, these include cell surface receptor signalling pathway (GO:0007166), cellular component morphogenesis (GO:0032989), cellular macromolecule localization (GO:0070727) and protein phosphorylation (GO:0006468).

### Epigenetic entropy

There was a significant interaction of chronological age and sex on their effects on epigenetic entropy (Beta regression: $\chi ^2$ = 5.4654, d.f. = 1, *p* = 0.0019) , see Fig. 2c. Females display the increasing pattern found in other species (F= 7.491, df = 1, *p* = 0.0062), but males display no relationship between entropy and chronological age (F= 0.304, df = 1, *p* = 0.5816).

### Epigenetic clock

The epigenetic clock was constructed by regressing chronological age against the 5290 significantly differentially methylated CpGs. This identified 19 CpGs that best predict age. Eight of these decrease in methylation as *Nasonia* age and eleven increase in methylation. The full list of these CpGs and the genes where they are located can be found in Supplemental Table S7. GO terms associated with these genes can be seen in Supplementary Fig. S6. These include glyoxylate cycle (GO:0006097), response to oxidative stress (GO:0006979), induction of programmed cell death (GO:0012502), and regulation of cell growth (GO:0001558).

The epigenetic age of each replicate is the weighted average of these CpGs’ methylation state. This correlates with chronological age (Spearman’s $\rho$ = 0.94, *p* = $1.4\times 10^{-8}$, see Fig. 2d), with a Root Mean Square Error of 0.39 days between chronological age and epigenetic age. Male and females show no difference in age acceleration (Wilcoxon test:W = 35, *p* = 0.9626), see Supplementary Fig. S7.

## Discussion

We have found evidence of dynamic DNA methylation changes associated with ageing in the model insect *Nasonia vitripennis*. This includes differentially methylated CpGs, variably methylated CpGs, an increase in epigenetic entropy in females and an epigenetic clock that accurately predicts age in *Nasonia*. Our methylation data is based on whole body samples. Individual cell or tissue types are known to have larger differences in methylation than are found throughout ageing [49]. Our finding of age-related epigenetic changes despite using whole body samples fits with the use of pan-tissue epigenetic clocks in humans, although see Porter et al. [49].

A limitation of this study is the short time span over which the methylation samples were collected. We based our BS-seq sampling on an earlier study that found that males had an average adult life expectancy of 10.7 days and females 16.6 days [48]. Our wasps lived 67% longer, meaning our methylation analysis captured a reduced amount of the ageing process. Despite this, our results still display clear ageing patterns in methylation. This fits with epigenetic clocks in other animals where there is a linear change in methylation over the course of the adult lifespan [27].

In our survivial experiment, females live longer than males. The survival curves also appear different. Females have the classic type 1 curve, but males appear almost as a type 3 curve with high mortality rates at a younger age, but the rate of death seems to slow down once males go past median lifespan. The increased lifespan of females reflects the pattern found in humans, most mammals and *Drosophila* [50]. In humans, this sex difference in lifespan is universal but in other species, it depends strongly on genotype and environment. Therefore, any general conclusions about sex difference in *Nasonia* ageing will need further experiments using different genotypes and environments.

Overall, DNA methylation increases in *Nasonia* as it ages. This is mirrored in the pattern of differentially methylated genes, with the majority being consistently hypermethylated as *Nasonia* age. This contrasts with the general hypomethylation found in mammalian ageing [51], although this is highly tissue specific with many tissues and cell types showing no relationship between global DNA methylation and ageing [5]. With the proviso that our data comes from whole body analysis, this hypermethylation pattern in ageing *Nasonia* might reflect the different roles of DNA methylation in invertebrates compared to mammals [52].

Females consistently exhibit lower levels of methylation compared to males as they age. Additionally, there is a much lower number of genes showing consistent hypermethylation or hypomethylation in aging females compared to aging males. This trend is reminiscent of a recent finding in the mealybug *Planococcus citri* [53], where males exhibit higher methylation levels distributed across the genome, while females have lower overall methylation but concentrated in specific regions. The authors hypothesize that this pattern is related to the mealybug’s paternal genome elimination process, wherein paternal chromosomes in males are highly condensed and eliminated from sperm. This sex-specific distribution of methylation may also be observed in *Nasonia*. Wang et al. [54] discovered that in *Nasonia*, only a few genes with male-biased expression are methylated, while a considerable number of genes with female-biased expression exhibit methylation.

Epigenetic drift, measured as either variably methylated positions or entropy increases as *Nasonia* ages. Nearly 8% of methylated CpGs were classified as variably methylated positions. Amongst them are a number of sirtuins (see Supplementary Table S6), which have been proposed as central to epigenetic ageing [55]. VMPs have been proposed to measure secondary ageing [5]. Primary ageing are changes in all tissues over time, secondary ageing are deleterious changes aggravated by the environment and disease [5]. This idea suggests that VMPs are the part of the methylome that responds to ageing interventions. That is the methylome becomes more youthful by returning to its less noisy original state.

Epigenetic entropy, at least in females, seems to follow the mammalian pattern, with the methylome becoming less predictable as female *Nasonia* age. A recent study showed that epigenetic entropy in a species predicts that species’ maximum lifespan [56]. Males, however show no relationship between chronological age and entropy. We offer two possible explanations of this. Firstly, the male pattern seems to be reflected in the survivourship curve (Fig. 2a), where our day 16 samples almost correspond to mean male lifespan (17 days). After this point, the rate of death seems to slow down. This slower rate of death might be associated with this decreased entropy in sixteen day old males, akin to the late life cessation of age-related deterioration found in many species [57]. Secondly, the lifespan of males appears more variable than females. This is most clearly seen in the age acceleration of the two sexes, see Supplementary Fig. S7. This increased variation in male lifespan could mean, with our small sample size, it is more difficult to find the relationship with entropy in males.

The tight correlation between chronological age and epigenetic age as calculated by the epigenetic clock is to be expected. There is very little variation in our test subjects. They all come from a single strain and are kept in exactly the same environment. That is, there is nothing to induce variation in biological ageing. We also found no age acceleration between males and females. This epigenetic clock is similar to results in many vertebrates [58] and even recently in the crustacean, the water flea *Daphnia magna* [9]. However, this is the first time an epigenetic clock has been discovered in a tractable insect model.

Unsurprisingly, there is no direct correspondence between the thirteen genes found in multiple human epigenetic clocks [59] and the genes associated with our nineteen clock CpGs. Although, the CpG having the most effect on epigenetic age in *Nasonia* is located in the gene for a leucine-rich repeat kinase (lrrk). LRRK2 mutations are a common cause of age related autosomal-dominant Parkinson’s disease [60]. Genes associated with neurodegenerative diseases are common in mammalian epigenetic clocks. There is some overlap in the GO terms associated with a pan-mammalian epigenetic clock [61] and those we found in *Nasonia* (Supplementary Fig. S6). These include nucleic acid binding and RNA metabolic processes.

We predict two main areas where our establishment of an epigenetic clock in a model insect species will be useful; firstly, the biology underpinning epigenetic clocks and secondly, how influenced epigenetic clocks are by ageing interventions. Variation in the rate of an individual’s epigenetic clock is affected by a large number of traits including inflammation, cell division, metabolic effects, cellular heterogeneity, diet, and numerous other lifestyle factors [26]. *Nasonia*, with its simplified insect systems, is perfect to experimentally separate the different processes involved in the biology of the clock into its constitutive parts [12]. Being short-lived (3-4 weeks as opposed to 26-30 months for mice), *Nasonia* are ideal to measure the effects of ageing interventions on both life span and epigenetic ageing. This will answer the question does a short-term decrease in someone’s epigenetic clock score lower their chance of developing age-related ill health, that is if epigenetic clocks can be used as endpoints for clinical trials of various anti-ageing interventions [28].

Starting with Medawar, genetic mutations were seen as the driver of ageing [62]. Recent theories on the causes of ageing focus rather on the loss of epigenetic information as the main driver of ageing [55]. These epigenetic factors due to their known plasticity, are tempting targets for anti-ageing interventions [63]. We propose *Nasonia vitripennis*, with its fully functional DNA methylation system and its now established ageing methylome as a model for this epigenetic era of ageing research.

### Supplementary Information

**Supplementary Material 1.**

## Data Availability

All sequencing data related to this project can be found under EBI ArrayExpress E-MTAB-13854324. Custom scripts fo the genome-wide analysis can be found at https://github.com/EamonnMallon/nasonia_326epigenetic_ageing.
