# Supplementary material for: Exploring the ageing methylome in the model insect, Nasonia vitripennis
Source: BMC Genomics. 2024 Mar 22;25:305. doi: 10.1186/s12864-024-10211-7 (PMC10958858; doi:10.1186/s12864-024-10211-7)
Supplement: Supplementary file 1 — Supplementary Material 1. [file 12864_2024_10211_MOESM1_ESM.zip › Exploring_the_ageing_methylome_in_the_model_insect__Nasonia_vitripennis_supplementary_file.pdf]

Supplemental file for:Exploring the ageing methylome in the model insect, *Nasonia vitripennis*.

K. Brink[1*], C.L. Thomas[1*], A. Jones[2], T.W. Chan[3] and E. B. Mallon[1†]

[1]Department of Genetics and Genome Biology, University of Leicester, University Road, Leicester, U.K.
[2]Institute for Evolution and Biodiversity, University of Muenster, Huefferstrabe, Muenster, Germany
[3]School of Life Sciences, Gibbet Hill Campus, The University of Warwick, Coventry, U.K.
[*]Equal contribution.
[†]Corresponding Author.

Friday 1st March, 2024

Running title: An insect model for the ageing methylome

Contact: ebm3@le.ac.uk

DRAFT

Supplementary figures and text

Supplementary Table S1: Differentially methylated genes associated with ageing in *Nasonia vitripennis*.
Supplementary Table S2: Consistently hypermethylated genes associated with ageing in male *Nasonia vitripennis*.
Supplementary Table S3: Consistently hypomethylated genes associated with ageing in male *Nasonia vitripennis*.
Supplementary Table S4: Consistently hypermethylated genes associated with ageing in female *Nasonia vitripennis*.
Supplementary Table S5: Consistently hypomethylated genes associated with ageing in female *Nasonia vitripennis*.
Supplementary Table S6: Genes containing variably methylated CpGs associated with ageing in *Nasonia vitripennis*.
Supplementary Table S7: CpGs making up the epigenetic clock in *Nasonia vitripennis*.
Supplementary Table S8: All differentially methylated CpGs with chromosomal positions and differences in methylation. Source refers to what comparision it came from, e.g. ”F016” is a comparison between day 0 females and day 16 females.

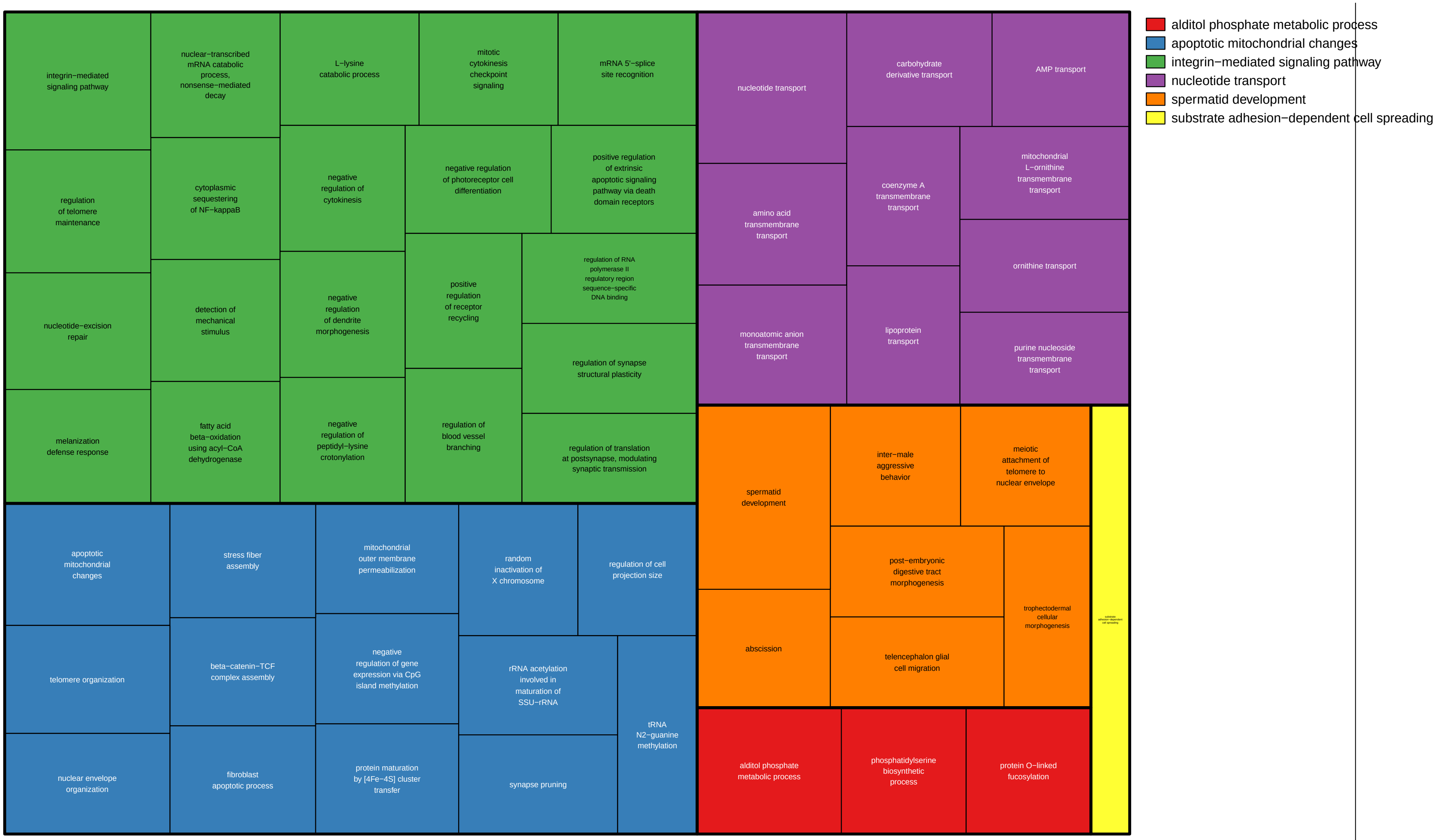

Figure S1: Treemap of Biological Processes GO term enrichment for consistently hypermethylated genes associated with ageing in males. Enriched BP for GO terms ($p < 0.05$) clustered using REVIGO. These rectangles are joined into different coloured 'superclusters' of loosely related terms. The area of the rectangles represents the p-value associated with that cluster's enrichment.

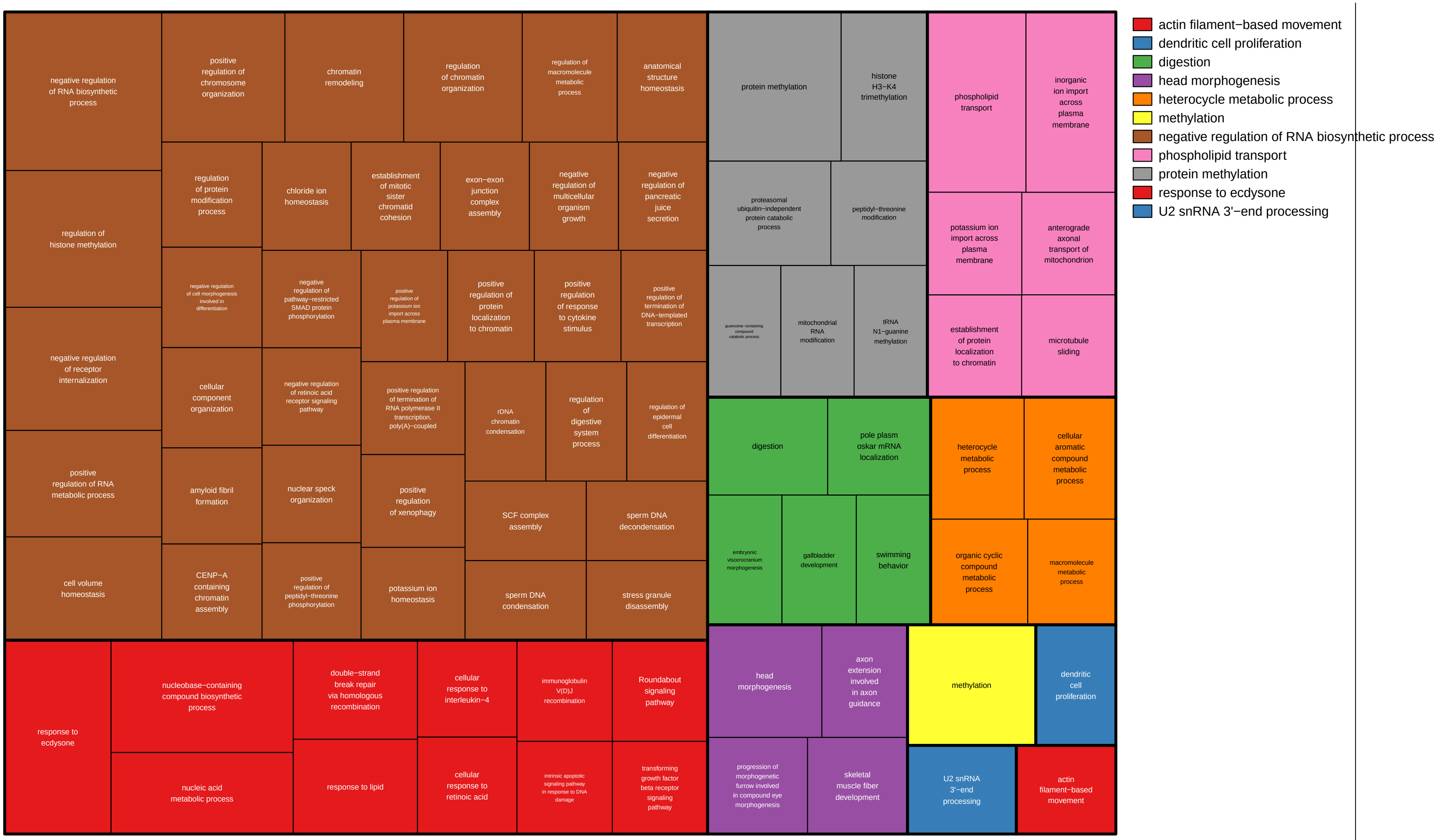

Figure S2: Treemap of Biological Processes GO term enrichment for consistently hypomethylated genes associated with ageing in males. Enriched BP for GO terms (p <0.05) clustered using REVIGO. These rectangles are joined into different coloured 'superclusters' of loosely related terms. The area of the rectangles represents the p-value associated with that cluster's enrichment.

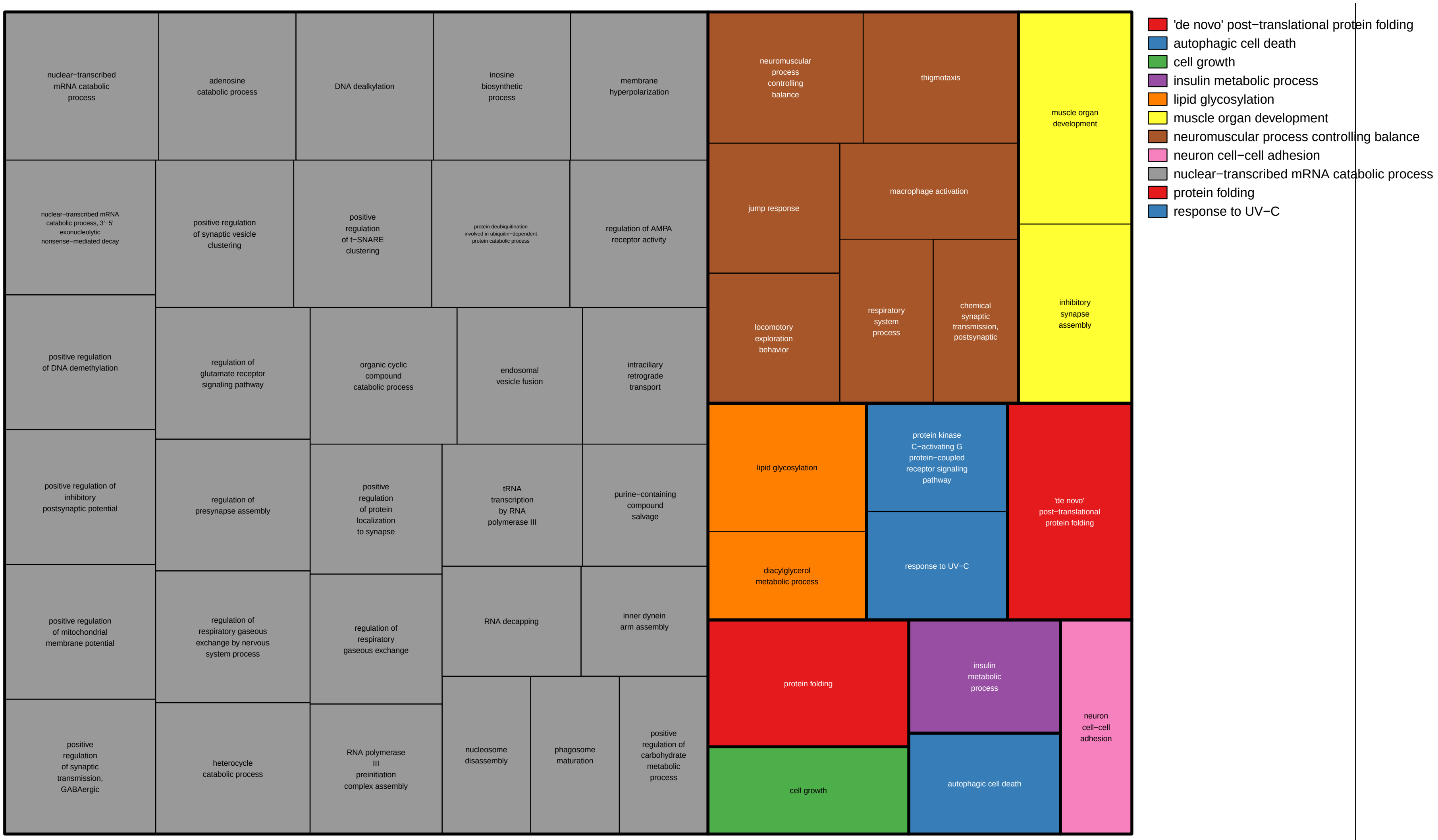

Figure S3: Treemap of Biological Processes GO term enrichment for consistently hypermethylated genes associated with ageing in females. Enriched BP for GO terms (p <0.05) clustered using REVIGO. These rectangles are joined into different coloured 'superclusters' of loosely related terms. The area of the rectangles represents the p-value associated with that cluster's enrichment.

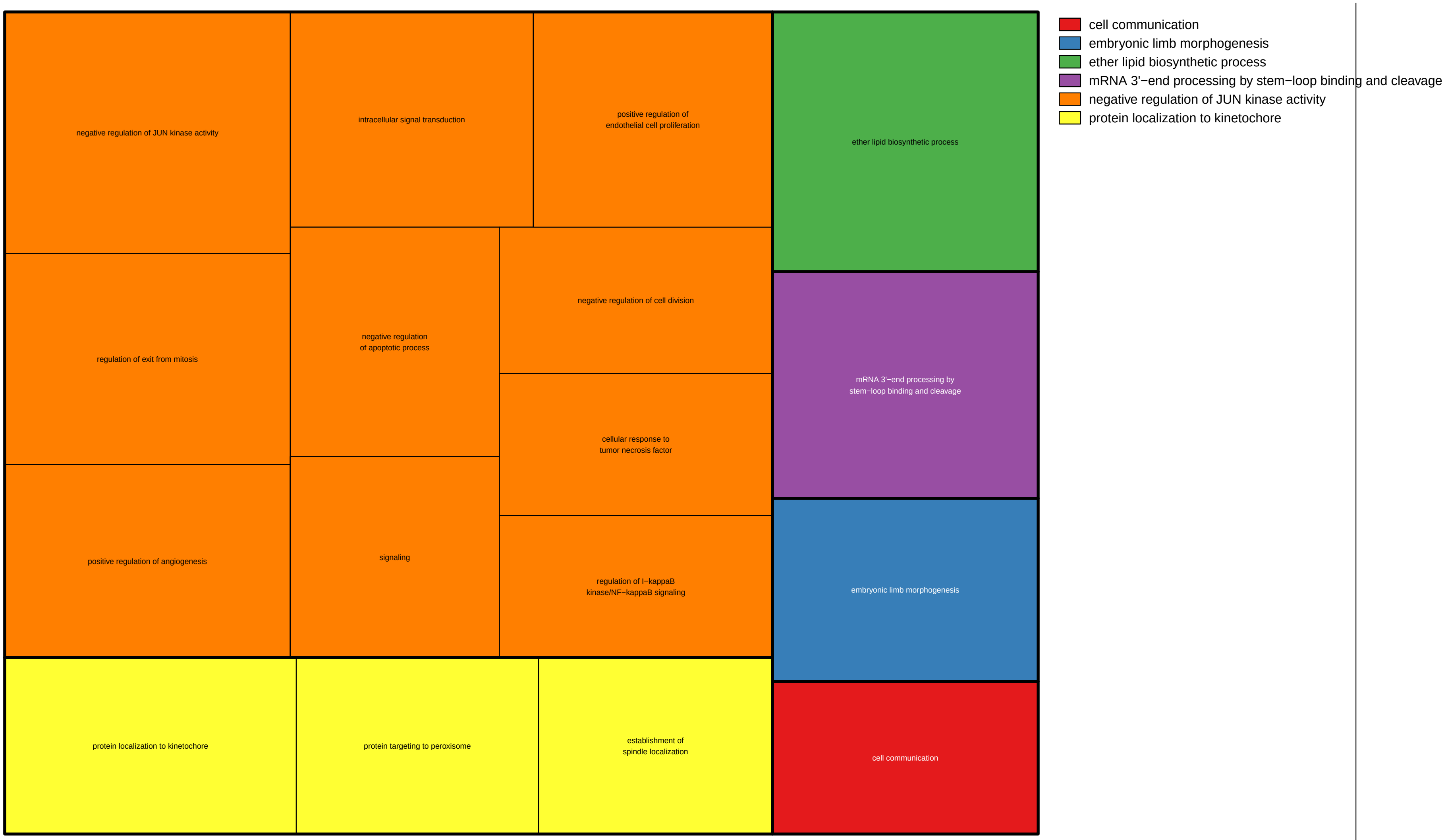

Figure S4: Treemap of Biological Processes GO term enrichment for consistently hypomethylated genes associated with ageing in females. Enriched BP for GO terms ($p < 0.05$) clustered using REVIGO. These rectangles are joined into different coloured 'superclusters' of loosely related terms. The area of the rectangles represents the p-value associated with that cluster's enrichment.

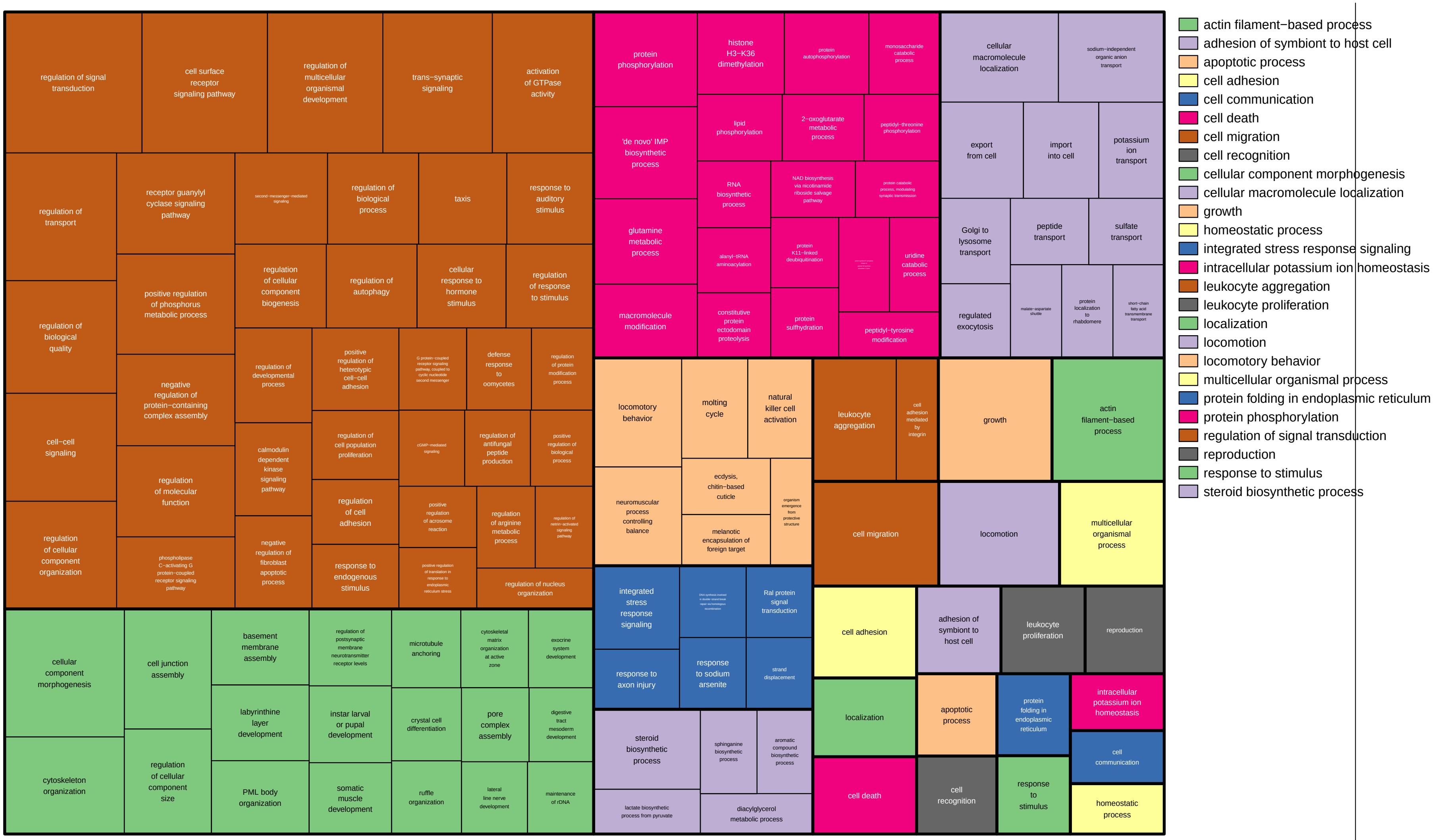

Figure S5: Treemap of Biological Processes GO term enrichment for variably methylated positions associated with ageing. Enriched BP for GO terms ($p < 0.05$) clustered using REVIGO. These rectangles are joined into different coloured 'superclusters' of loosely related terms. The area of the rectangles represents the p-value associated with that cluster's enrichment.

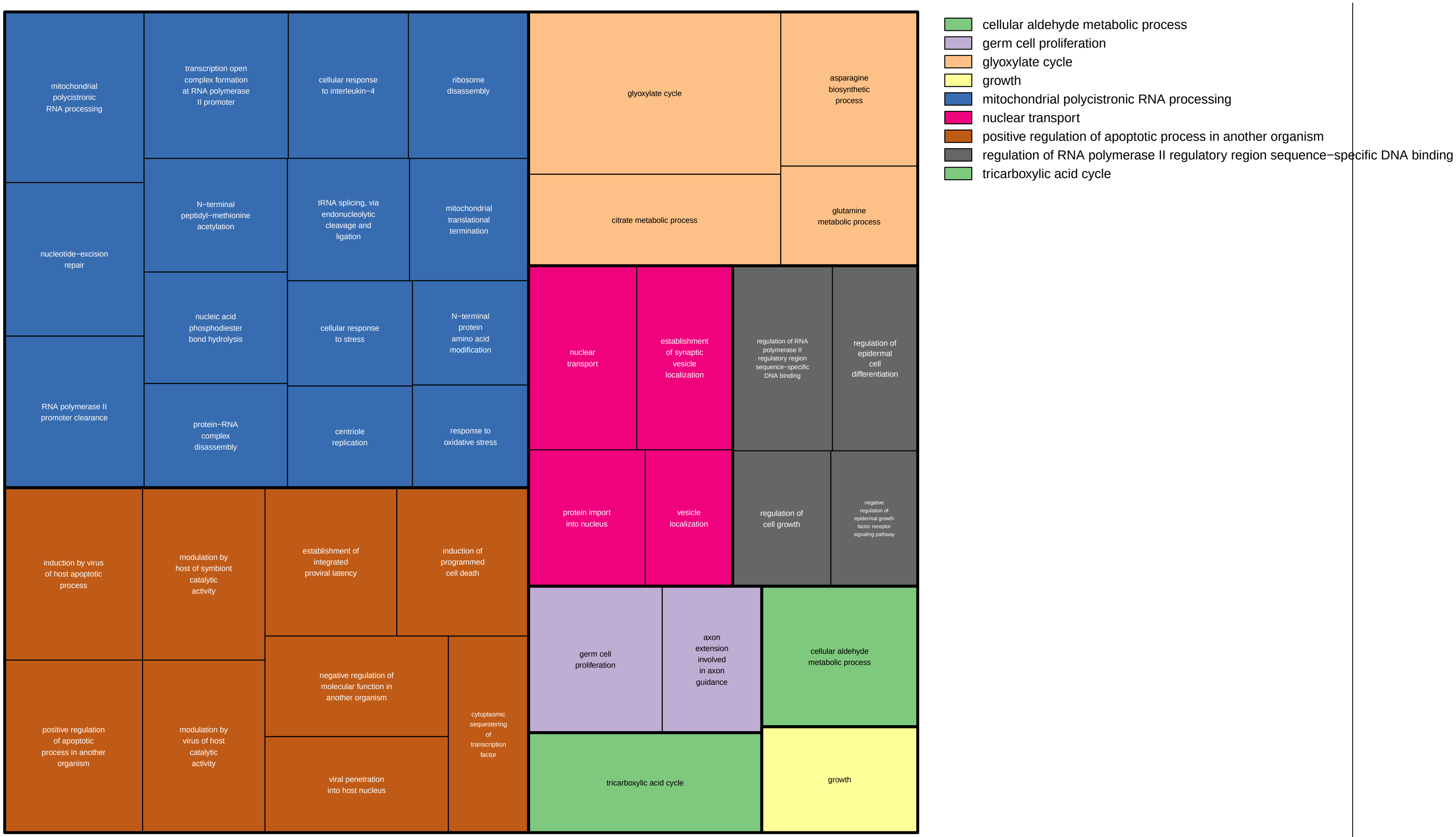

Figure S6: Treemap of Biological Processes GO term enrichment for epigenetic clock genes associated. Enriched BP for GO terms ($p < 0.05$) clustered using REVIGO. These rectangles are joined into different coloured ‘superclusters’ of loosely related terms. The area of the rectangles represents the p-value associated with that cluster’s enrichment.

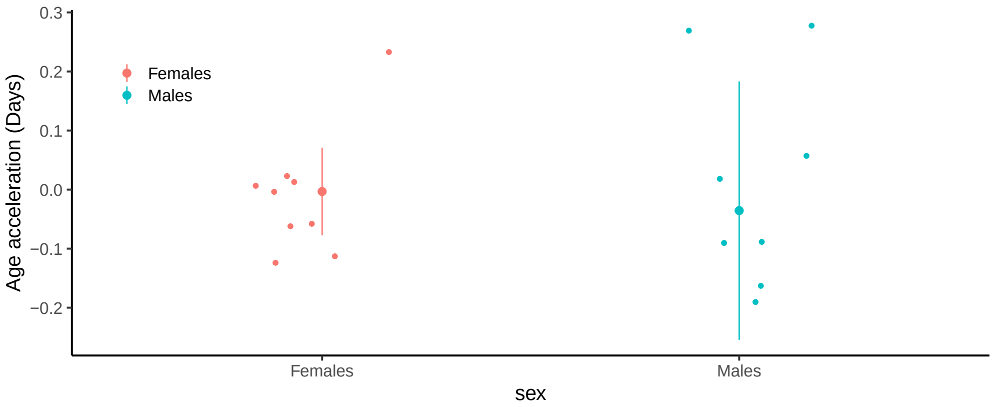

Figure S7: Age acceleration calculated as the residual of chronological age against epigenetic age for each sample. The large central dots represent the median value, with the vertical lines representing interquartile range.
